# Non-Invasive Capnography Versus Pulse Oximetry for Early Detection of Respiratory Depression During Pediatric Procedural Sedation: A Prospective Observational Study

**DOI:** 10.3390/children12070938

**Published:** 2025-07-16

**Authors:** Laura Català Altarriba, Sean Yeh Hsi, Aude Marie Ravit, Sònia Brió Sanagustín, Xoan González-Rioja

**Affiliations:** Hospital de la Santa Creu i Sant Pau, 08025 Barcelona, Spain; lcatala@santpau.cat (L.C.A.); aravit@santpau.cat (A.M.R.); sbrio@santpau.cat (S.B.S.); xgonzalezr@santpau.cat (X.G.-R.)

**Keywords:** pediatric sedation, capnography, respiratory monitoring, respiratory depression, procedural sedation

## Abstract

Background/Objectives: Continuous ventilation monitoring during pediatric sedation is essential, as respiratory depression may occur silently and may not be detected promptly by conventional methods such as pulse oximetry. Non-invasive capnography has been proposed to improve early detection of respiratory compromise. This prospective observational study evaluated the diagnostic accuracy of non-invasive capnography, compared to pulse oximetry, for detecting respiratory depression in pediatric patients undergoing sedation. Methods: We conducted a single-center, prospective observational study at a tertiary pediatric hospital, enrolling 101 patients (ages 1–17 years) undergoing sedation for diagnostic or therapeutic procedures. Patients were monitored using both pulse oximetry and non-invasive capnography. Episodes of respiratory depression—defined as apnea, hypopneic hypoventilation, bradypneic hypoventilation, and desaturation—were recorded. We compared the diagnostic performance and time to detection between capnography and pulse oximetry. Results: We identified 93 episodes of respiratory depression in 52 patients (51.1%). Capnography detected all apnea episodes and 76.9% of hypopneic hypoventilation episodes that were not identified by pulse oximetry. The median time advantage of capnography over pulse oximetry was 35 s (*p* = 0.0055). Combining capnography and pulse oximetry identified more events than pulse oximetry alone (93 vs. 53 episodes). Conclusions: Non-invasive capnography improves the early detection of respiratory depression compared to conventional monitoring with pulse oximetry in pediatric procedural sedation. While these findings support its routine use to enhance patient safety, larger multicenter studies are needed to demonstrate its diagnostic accuracy and impact on clinical outcomes.

## 1. Introduction

Procedural sedation and analgesia (PSA) is increasingly employed in pediatric emergency and hospital settings to facilitate diagnostic and therapeutic procedures by minimizing pain, anxiety, and movement. However, sedation poses inherent risks, particularly respiratory depression, due to the combined physiological and pharmacological vulnerability of children. Ensuring patient safety during PSA requires vigilant monitoring to detect early signs of respiratory compromise [1,2,3,4].

The American Society of Anesthesiologists (ASA) and the Joint Commission on Accreditation of Healthcare Organizations define four levels of sedation based on the depth of consciousness depression and physiological function impairment [1,2,5]

- Minimal sedation preserves normal responses to verbal commands, with minor cognitive impairment and no impact on ventilatory or cardiovascular function.
- Moderate sedation allows purposeful responses to verbal or tactile stimuli, with spontaneous ventilation and hemodynamic stability, without requiring airway intervention.
- Deep sedation is characterized by a response only to repeated or painful stimuli, potential ventilatory compromise requiring assistance, and usually preserved cardiovascular function.
- General anesthesia induces complete unconsciousness, with no response to painful stimuli, impaired spontaneous ventilation requiring support, and possible cardiovascular depression.

This classification guides the safe and appropriate administration of sedation and anesthesia in clinical practice [1,2,4]. To minimize adverse outcomes, current guidelines recommend continuous monitoring, including pulse oximetry, electrocardiography, and direct clinical observation, supported by personnel trained in advanced resuscitation and pharmacologic management [1,2,3,6,7].

While pulse oximetry remains a cornerstone of oxygenation monitoring, it has critical limitations: it reflects oxygen saturation with a delay of up to 2–3 min, especially when supplemental oxygen is used, and it does not directly monitor ventilation [1,3,4,6,7,8,9,10,11,12,13,14,15,16,17,18,19].

In contrast, non-invasive capnography enables continuous, real-time monitoring of ventilation by measuring end-tidal carbon dioxide (EtCO_2_), allowing for earlier detection of hypoventilation and apnea, before oxygen desaturation is observed [1,4,6,7,9,10,11,12,13,14,18,20,21,22,23]. EtCO_2_ provides both a graphical waveform and a numerical value, with excellent correlation with arterial carbon dioxide (PaCO_2_) [9,10,18,24], with a typical difference of 2–5 mmHg in healthy individuals. In pediatric patients, normal EtCO_2_ values generally range from 35 to 40 mmHg [4,6,8,13,14,15,21,23,24,25]. This close correlation makes capnography an essential tool for assessing ventilatory status during sedation and is reliable in low perfusion states [8].

Reflecting its clinical value, current guidelines recommend incorporating capnography during moderate to deep sedation to enhance patient safety [1,2,6,9]. Nevertheless, its implementation remains inconsistent: a recent multicenter survey found that only 46% of pediatric emergency departments across Europe routinely utilize capnography during procedural sedation [26].

Although studies in adults have demonstrated that capnography enhances early detection of respiratory compromise, evidence in pediatric populations remains limited. Moreover, the comparative diagnostic accuracy and time advantage of capnography over pulse oximetry in identifying specific respiratory events—such as apnea or hypoventilation—require further investigation [1,4,7,10,11,12].

Among the available techniques, sidestream capnography is particularly well suited for non-intubated pediatric patients. It samples exhaled air through nasal cannulas and avoids bulky airway attachments required by mainstream systems. Sidestream devices are less invasive, adaptable to varied clinical settings, and reduce infection risk through disposable components, making them ideal for pediatric monitoring [8,13,14,17,21,25].

Early indicators of respiratory depression include a decrease in respiratory rate (RR) or a rise in EtCO_2_. Capnography also facilitates the detection of complications such as upper airway obstruction, laryngospasm, and bronchospasm, enabling timely intervention to prevent severe outcomes [3,6,8,9,12,13].

The aim of this study was to assess the diagnostic accuracy of non-invasive capnography for the early detection of respiratory depression in pediatric patients undergoing procedural sedation, compared to conventional pulse oximetry. Secondary objectives included characterizing the types of respiratory events detected and analyzing the temporal relationship between capnographic and oximetric detection, with the goal of evaluating the clinical relevance of early identification.

## 2. Materials and Methods

### 2.1. Study Design

We conducted a prospective, single-center observational study at a tertiary care pediatric hospital between October 2022 and June 2024. The study aimed to assess the diagnostic accuracy of non-invasive capnography, compared to conventional pulse oximetry, for the early detection of respiratory depression during procedural sedation. Ethical approval was obtained from the institutional review board, and informed consent was secured from the parents or legal guardians of all participants.

### 2.2. Participants

Pediatric patients aged 1 to 17 years undergoing diagnostic or therapeutic procedures requiring sedation were consecutively enrolled. Inclusion required signed informed consent and eligibility under institutional sedation protocols.

Exclusion Criteria

The exclusion criteria can be seen in Table 1.

### 2.3. Sedation and Monitoring Protocol

Sedation was administered by the institutional pediatric sedation team in accordance with standardized protocols. Sedative regimens typically included combinations of propofol, ketamine, midazolam, and/or fentanyl, selected based on the specific procedure and individual patient characteristics.

An attending pediatrician supervised all sedations, while intravenous medications were administered by a trained pediatric emergency department nurse. When appropriate, additional specialists—such as orthopedic surgeons during fracture reductions—were present to perform or support procedural interventions.

#### 2.3.1. Monitoring

Patients were continuously monitored from immediately before the start of sedation until the completion of the procedure. This included the following:

- Pulse oximetry (SpO_2_) for oxygenation;
- Non-invasive, self-calibrating sidestream capnography (GE Healthcare E-miniC-00, GE Healthcare, White Marsh, MD, USA) via nasal cannulas (Natus^®^, Natus Medical Incorporated, Middleton, WI, USA) for EtCO_2_ measurement and respiratory rate monitoring;
- Standard vital signs, including heart rate and blood pressure.

SpO_2_, EtCO_2_, respiratory rate, and heart rate were recorded every 10 s, and blood pressure was recorded every 5 min. Data collection ended when the procedure was completed.

#### 2.3.2. Evaluation Scales

Several scales were used to assess sedation levels and pain during the procedures:

- Ramsay Sedation Scale to assess sedation level. Sedation levels were measured at minute 0, and every 5 min until the end of the procedure.
- FLACC Scale, Wong-Baker Faces Scale, and Visual Analog Scale (VAS) were used, depending on the patient’s age, to assess pain during the procedure.

### 2.4. Definition of Respiratory Events

Respiratory depression events were defined as follows:

- Apnea: Absence of breathing or capnographic waveform for more than 2 consecutive readings.
- Hypopneic Hypoventilation: EtCO2 < 30 mmHg or a decline greater than 10 mmHg from baseline with normal or decreased respiratory rate for more than 2 consecutive readings. It detects a decrease in the tidal volume while the patient is maintaining a normal respiratory rate, which leads to EtCO2 levels that are lower than normal, mainly because of a relative increase in dead space ventilation.
- Bradypneic Hypoventilation: Decreased respiratory rate with EtCO2 > 50 mmHg for more than 2 consecutive readings with an abnormally low respiratory rate per age.
- Desaturation: SpO2 < 95% measured by pulse oximetry for more than 2 consecutive readings.
- Respiratory rate (RR) [27] per age was defined as follows:
  ○ 1–2 years: 20–40 breaths per minute (bpm);
  ○ 2–8 years: 20–30 bpm;
  ○ 8–12 years: 15–25 bpm;
  ○ >12 years: 12–24 bpm.

These definitions were consistent with published pediatric sedation monitoring standards [6,8,15,16].

In the absence of a universally standardized time threshold in clinical guidelines, we defined respiratory depression events using a 2-consecutive-reading criterion, aligning with commonly accepted practice in sedation research. This interval was selected based on two main considerations:

(1) Our monitoring system recorded vital parameters every 10 s, making 2 consecutive readings the shortest practical duration to confirm a sustained abnormality; and
(2) Clinical relevance, as transient deviations shorter than this period are often self-resolving and may not warrant intervention.

While major guidelines recommend continuous capnographic monitoring during moderate-to-deep sedation, they do not specify a minimum duration to define apnea or hypoventilation events [1]. However, several sedation studies—including in pediatric populations—have operationalized apnea or hypoventilation as the absence or significant reduction in respiratory activity for ≥15 s based on capnography. Many capnography devices also use similar thresholds to trigger apnea alarms [4,12,23].

### 2.5. Sample Size and Power Analysis

The sample size was calculated to detect significant differences in the frequency of respiratory depression events between monitoring modalities (capnography vs. pulse oximetry), based on an anticipated desaturation rate of 10%. However, more recent studies—such as Lee et al. (2025)—have reported higher event rates (up to 27.6%), suggesting that the original estimate may have been conservative [28].

A total of 101 patients were ultimately enrolled, with the final sample size constrained by patient availability and resource limitations over the study period. While this cohort was sufficient to address the primary hypothesis regarding detection timing, the sample was not specifically powered to evaluate diagnostic accuracy parameters such as sensitivity, specificity, or predictive values. Consequently, this limits the precision of comparative performance estimates between the monitoring methods and restricts the robustness of subgroup analyses across demographic or clinical strata.

### 2.6. Statistical Analysis

Statistical analyses were performed using SPSS version 17.0 (IBM Corp., Armonk, NY, USA). Descriptive statistics summarized patient demographics and clinical outcomes. For the subset of respiratory events detected by both capnography and pulse oximetry, we evaluated the difference in detection times using a one-sided Wilcoxon signed-rank test.

This non-parametric test was chosen due to the small sample size and non-normal distribution of time differences. A one-sided test was applied based on the a priori clinical expectation that capnography, which provides direct real-time ventilation data, would detect respiratory depression earlier than pulse oximetry, which reflects downstream oxygenation changes with inherent delay. This directional hypothesis justified the use of a one-sided approach to improve statistical power while maintaining control over type I error. Statistical significance was defined as *p* < 0.05.

In the subset of 21 episodes where both methods identified events, we calculated the time difference between capnographic and oximetric detection. We also explored the association between ASA physical status and the occurrence of respiratory events using chi-square and Spearman correlation tests.

## 3. Results

### 3.1. Patient Characteristics

A total of 101 pediatric patients were included between October 2022 and June 2024. Table 2 summarizes patient demographics.

The procedures performed included a wide range of interventions, and the full list of procedures is provided in Table 3. These were performed both in the emergency department and other areas of the hospital.

### 3.2. Sedation and Pain Scores

- Ramsay sedation scale: Sedation depth was assessed using the Ramsay scale. At baseline (minute 0), the median score was 2 (range 1–3), indicating that patients were calm and cooperative. By minute 5, the median increased to 4 (range 3–5) and remained stable through minute 30, reflecting sustained moderate to deep sedation.
- Age-adjusted pain scales (FLACC, Wong-Baker Faces, or VAS): median pain score 0 during the first 15 min, slightly increasing to a median of 1 from minute 20 onward.

### 3.3. Respiratory Depression Events

A total of 93 respiratory depression events were recorded in 52 patients (51.1%). View Table 4.

#### 3.3.1. Hypopneic Hypoventilation: 52 Episodes in 35 Patients

- Twelve events (23.0%) associated with desaturation.

#### 3.3.2. Isolated Bradypneic Hypoventilation: 0 Episodes

- No events met the capnographic criteria for bradypneic hypoventilation.

#### 3.3.3. Isolated Desaturation: 32 Episodes in 21 Patients

- Twenty-seven episodes with saturation 90–94%;
- Five episodes with saturation 85–89%;
- No episodes below 85%.

#### 3.3.4. Apnea: 9 Episodes, All Detected Early by Capnography

- Median delay before detection by pulse oximetry: 10 s (range 0, +40 s);
- Lowest observed saturation: 60%, median 85% (interquartile range 79–91%).

A total of nine apneas were observed in seven patients, each exhibiting distinct physiological patterns.

- Three apneas began with a decrease in end-tidal CO_2_ (EtCO_2_), followed by a cessation of respiratory rate (RR = 0), and ended with oxygen desaturation.
- One apnea was characterized by an initial drop in EtCO_2_, followed by oxygen desaturation, and concluded with a cessation of respiratory rate (RR = 0), which had previously been abnormally low.
- One apnea presented an initial increase in EtCO_2_, consistent with bradypneic hypoventilation, followed by oxygen desaturation and subsequent cessation of respiratory rate (RR = 0).
- The remaining four apneas showed a simultaneous drop in EtCO_2_, respiratory rate, and oxygen saturation.

### 3.4. Time Difference Analysis

Among the 21 episodes in which both capnography and pulse oximetry detected respiratory events, capnography identified the event a median of 35 s earlier than pulse oximetry (95% confidence interval: 20–60 s), with an interquartile range of 42.5 s and a range of 190 s. In 13 of these 21 episodes (61.9%), capnography detected the respiratory event before pulse oximetry. In five episodes (23.8%), both modalities detected the event simultaneously, while in only three episodes (14.3%) did pulse oximetry detect the event before capnography.

A one-sided Wilcoxon signed-rank test was conducted to test whether capnography detected events significantly earlier than pulse oximetry (i.e., whether the median time difference was greater than zero). The test rejected the null hypothesis (test statistic = 116.0, *p* = 0.0055), indicating a statistically significant lead in detection time for capnography.

**Figure 1:**
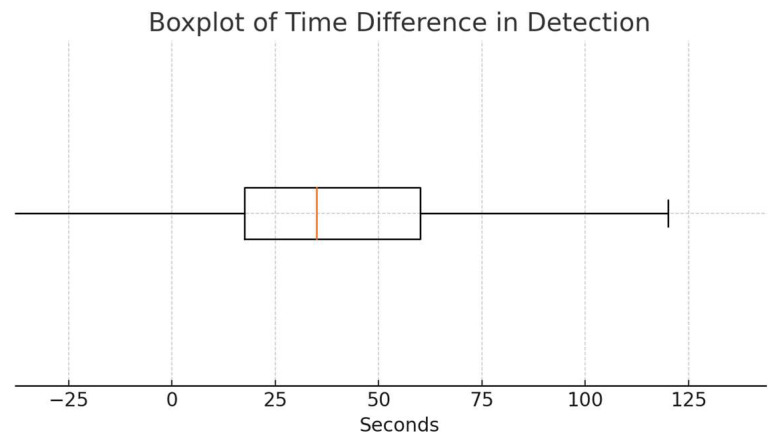
Boxplot showing the distribution of time differences (in seconds) between capnographic and oximetric detection of respiratory events. Positive values indicate that capnography detected events earlier than pulse oximetry.

Figure 1 presents a boxplot illustrating the distribution of time differences between the two detection methods.

### 3.5. Clinical Interventions

Most respiratory events resolved spontaneously without requiring intervention. When intervention was needed, the most common measures were supplemental oxygen administration, airway repositioning, and bag-mask ventilation. No cases required endotracheal intubation.

### 3.6. ASA Classification

Of the included patients, 71% were classified as ASA 3 (mainly oncologic patients), compared to 29% classified as ASA 1 or 2. Statistical analysis showed no significant association between ASA classification and the occurrence of respiratory events (chi-square 2.28, *p* = 0.131) or the time difference in detection between capnography and pulse oximetry (Spearman coefficient −0.023, *p* = 0.922).

## 4. Discussion

This prospective observational study evaluated the diagnostic accuracy of non-invasive capnography compared to pulse oximetry for detecting respiratory depression in pediatric patients undergoing procedural sedation. The key finding was that capnography detected respiratory events significantly earlier, with a median time advantage of 35 s (95% CI: 20–60 s), particularly in episodes of hypopneic hypoventilation and apnea.

While apnea and bradypnea can be identified through a decrease in respiratory rate, hypopneic hypoventilation—characterized by a decrease in tidal volume—is more difficult to detect through visual inspection or conventional monitoring. This diagnostic challenge may lead to adverse effects such as hypoxia or apnea [6]. In our study, 12 patients with hypopneic hypoventilation subsequently developed desaturation, highlighting its potential value as a predictor of respiratory deterioration [4,6,7,18].

These findings are consistent with prior studies in both adult and pediatric cohorts, which have demonstrated that capnography confers superior sensitivity for early detection of hypoventilation relative to pulse oximetry [3,4,6,7,10,13,15,16,23]. Notably, in our study, 76.9% of hypopneic hypoventilation episodes were not accompanied by desaturation, underscoring the limitations of relying solely on oxygen saturation as a surrogate for ventilatory status.

The clinical relevance of early detection cannot be overstated in pediatric populations, where limited respiratory reserve means that even short delays in recognizing ventilatory compromise may lead to adverse outcomes. Although most events in this cohort resolved spontaneously, timely identification allows clinicians to anticipate deterioration, adjust sedation depth, or intervene with airway maneuvers before hypoxemia develops [3,4,6,7,9,10,17,18].

It is important to note that oxygenation and ventilation are not always correlated. Alveolar hypoventilation can occur despite normal SpO_2_ values, resulting in elevated EtCO_2_ levels without desaturation [9,14]. In our study, four patients presented EtCO_2_ values exceeding 50 mmHg. Of these, only one exhibited an abnormally low respiratory rate, which subsequently progressed to apnea. The remaining three episodes showed EtCO_2_ >50 mmHg but maintained normal to elevated respiratory rates and thus did not meet criteria for bradypneic hypoventilation.

As previously mentioned, hypoxia is a late finding in respiratory depression. The relationship between PaO_2_ and SpO_2_ is not linear, allowing significant changes in oxygenation to go unnoticed before being detected by pulse oximetry. In the study by Yildizdas et al., 21 episodes of respiratory depression were detected, with hypoxia present in 3.2% (4 patients) and hypercarbia in 16.6% (17 patients). If only pulse oximetry had been used, only four of these episodes would have been identified [18].

In our study, the exclusive use of pulse oximetry would have allowed detection of only 53 episodes of respiratory depression, compared to the 93 identified through the combined use of pulse oximetry and capnography. A 2017 meta-analysis demonstrated that the combination of capnography, direct visual inspection, and pulse oximetry reduced the incidence of both moderate desaturation [risk ratio (RR) 0.77, 95% CI 0.67–0.89] and severe desaturation (RR 0.59, 95% CI 0.43–0.81), in addition to lowering the need for assisted ventilation during procedural sedation performed outside a pediatric intensive care unit [29].

Regarding the 32 episodes of isolated desaturation without concurrent alterations in the capnography record, these may be attributable to oxygenation-specific phenomena that occur independently of changes in respiratory rate or EtCO_2_. Potential contributors include patient movement, hypothermia, or the presence of dyshemoglobinemias. Additionally, pulse oximetry has an inherent margin of error of approximately ±2% when compared to invasive methods of oxygen saturation monitoring [30]. It is also important to note that our definition of desaturation was particularly strict, considering values ≤ 94% as events. This conservative threshold may have led to an overestimation of the true number of clinically significant desaturation episodes.

In relation to the nine apnea episodes, these findings underscore the heterogeneous presentation of apnea and highlight the utility of continuous EtCO_2_ monitoring for early detection of respiratory compromise. In five cases, changes in EtCO_2_ preceded alterations in respiratory rate and oxygen saturation, suggesting that capnography provides earlier warning of impending respiratory compromise. However, because physiological parameters were recorded at 10 s intervals, subtle differences in onset timing shorter than that interval may have gone unnoticed. This is particularly relevant in the four apneas where EtCO_2_, respiratory rate, and oxygen saturation changed simultaneously; in these instances, we cannot determine which signal truly changed first. While it is possible that EtCO_2_ detected the event earlier, this may have escaped documentation due to the resolution limits of the data.

The effects of intermittent hypoxia on the developing brain in children are not fully understood. However, both chronic and intermittent hypoxia have been shown to negatively impact development, behavior, and academic performance [4,6,16,20,31]. Therefore, the incidence of these events should be minimized.

Importantly, no significant association was found between ASA classification and either the frequency or timing of respiratory events. This suggests that the benefit of capnographic monitoring extends across patient risk profiles, including the high proportion of ASA III (mainly oncologic) patients represented in this study.

### 4.1. Study Limitations

Several limitations warrant acknowledgement:

- Single-center design: Conducted in a tertiary pediatric hospital, limiting generalizability to other settings.
- Sample size and estimation approach: Although the sample (*n* = 101) was adequate for primary analyses, it limited subgroup comparisons (e.g., by age or comorbidities). Additionally, the sample size calculation was based on clinical trial assumptions rather than diagnostic accuracy frameworks.
- Selection bias: Given that this study was conducted in a major oncology center, the high proportion of oncologic patients (ASA III) may influence the frequency of respiratory events, although no statistical association was found.
- Monitoring and clinical decision-making: Although capnography and pulse oximetry were both used for monitoring, clinical decisions during procedures (e.g., adjustments in sedation, oxygen administration, or airway interventions) were based solely on pulse oximetry and standard clinical assessment.
- Our primary objective was to compare the timing of respiratory event detection between capnography and pulse oximetry, and thus, the study was not prospectively designed to capture detailed intervention data. As such, the number of cases requiring clinical interventions, including supplemental oxygen administration, airway repositioning, or bag-mask ventilation, was not systematically documented. However, correlating earlier detection with actual clinical interventions would provide critical insight into the practical significance of our findings.
- Equipment limitations: Use of nasal cannulas (rather than nasobuccal cannulas) may have underestimated EtCO_2_ values in cases of intermittent oral breathing. Sidestream capnography is limited by a smaller amount of gas analyzed, has a slower response time than microstream capnography, and there is a higher risk of gas mixing [14,21,32]. Although we recognize the importance of the capnography waveform, our monitoring system did not have the capability to store waveform tracings for later analysis. As a result, we collected data in real time during sedation, recording only numerical values. Consequently, it was not possible to perform a detailed morphological analysis of the four phases of the capnogram or to explore relationships between waveform characteristics, patient gender, or procedural complexity.

### 4.2. Clinical Implications and Future Directions

Our findings support incorporating non-invasive capnography alongside conventional monitoring during pediatric procedural sedation to improve early detection of respiratory depression. This may enhance patient safety by enabling timely clinical interventions, especially in resource-constrained settings where visual monitoring may be limited.

However, larger multicenter studies are needed to validate these results, refine diagnostic accuracy metrics (sensitivity, specificity, predictive values), and evaluate the impact of capnography-guided interventions on patient outcomes, including procedural success and complication rates.

## 5. Conclusions

This study showed that non-invasive capnography detects respiratory depression earlier than pulse oximetry during pediatric procedural sedation, identifying events that might otherwise go unnoticed. Although clinical decisions in this study relied exclusively on conventional monitoring, our findings underscore the incremental value of incorporating capnography to enhance early detection of respiratory compromise. Future multicenter research is needed to confirm these results and assess their impact on clinical outcomes.

## Figures and Tables

**Table 1:** Exclusion criteria.

Exclusion Criteria
Emergency situations Patients with abnormal baseline EtCO_2_ or oxygen saturation (O_2_ Sat.) ^1^
Intubation
Patients with liver or kidney disease
Patients requiring home oxygen therapy
Intolerance to nasal cannulas
Injuries to the choanae or nasal mucosa
Difficult intubation predictors (craniofacial malformations, short neck, or Mallampati score 3–4)
History of severe allergic reaction to sedative drugs

^1^ e.g., bronchospasm, diabetic ketoacidosis, severe dehydration.

**Table 2:** Demographic information.

Demographic Information	
Age	Median 10.57 years (Interquartile range: 1.07 to 17.91 years)
ASA	Median 3 (Interquartile range: 1 to 3)
Fasting time	Median 11.30 h (Interquartile range: 3 to 28 h)
Sex	47 boys
	54 girls

**Table 3:** Procedures performed.

Procedure	Count
Lumbar puncture	33
Bone marrow aspiration	32
Fracture reduction	9
Bone marrow aspiration and lumbar puncture	6
Central venous catheter placement	4
Digestive endoscopy	4
Peripherally inserted central catheter (PICC) placement	3
Abscess drainage	2
Skin biopsy	1
Molluscum contagiosum cryotherapy	1
Pneumothorax drainage	1
Fecaloma extraction	1
Magnetic resonance imaging (MRI)	1
Urinary catheterization	1

**Table 4:** Respiratory depression events.

Respiratory Depression Events	Capnography	Pulse Oximetry
Hypopneic Hypoventilation	52	12 *
Bradypneic Hypoventilation	0	0
Apnea	9	9
Isolated Desaturation	-	32

* Hypopneic hypoventilation is detected exclusively by capnographic findings. However, among the 52 episodes identified, 12 were also associated with oxygen desaturation detectable by pulse oximetry.

## Data Availability

The data supporting the findings of this study are available from the corresponding author upon reasonable request. The data are not publicly available due to privacy concerns, as they include personally identifiable information such as dates of birth and procedure dates that could compromise patient confidentiality.

## Abbreviations

The following abbreviations are used in this manuscript:

PSA	Procedural Sedation and Analgesia Multidisciplinary Digital Publishing Institute
EtCO_2_	End-tidal Carbon Dioxide
SpO_2_	Peripheral Oxygen Saturation (by pulse oximetry)
ASA	American Society of Anesthesiologists Physical Status Classification
IQR	Interquartile Range
VAS	Visual Analog Scale
FLACC	Face, Legs, Activity, Cry, Consolability (pain scale)
CI	Confidence Interval

## References

[1] Coté C.J., Wilson S., American Academy of Pediatrics, American Academy of Pediatric Dentistry (2019). Guidelines for Monitoring and Management of Pediatric Patients Before, During, and After Sedation for Diagnostic and Therapeutic Procedures. Pediatrics.

[2] American Society of Anesthesiologists Task Force on Sedation and Analgesia by Non-Anesthesiologists (2002). Practice guidelines for sedation and analgesia by non-anesthesiologists. Anesthesiology.

[3] Mora Capín A., Míguez Navarro C., López López R., Marañón Pardillo R. (2014). Utilidad de la capnografía en la monitorización durante procedimientos de sedoanalgesia. Influencia de la administración de oxígeno en los parámetros monitorizados [Usefulness of capnography for monitoring sedoanalgesia: Influence of oxygen on the parameters monitored]. An. Pediatría.

[4] Lightdale J.R., Goldmann D.A., Feldman H.A., Newburg A.R., DiNardo J.A., Fox V.L. (2006). Microstream capnography improves patient monitoring during moderate sedation: A randomized, controlled trial. Pediatrics.

[5] The Joint Commission (2021). Comprehensive Accreditation Manual for Hospitals (CAMH): The Official Handbook.

[6] Langhan M.L., Chen L., Marshall C., Santucci K.A. (2011). Detection of hypoventilation by capnography and its association with hypoxia in children undergoing sedation with ketamine. Pediatr. Emerg. Care.

[7] Burton J.H., Harrah J.D., Germann C.A., Dillon D.C. (2006). Does end-tidal carbon dioxide monitoring detect respiratory events prior to current sedation monitoring practices?. Acad. Emerg. Med..

[8] Tobias J.D. (1999). End-tidal carbon dioxide monitoring during sedation with a combination of midazolam and ketamine for children undergoing painful, invasive procedures. Pediatr. Emerg. Care.

[9] Miner J.R., Heegaard W., Plummer D. (2002). End-tidal carbon dioxide monitoring during procedural sedation. Acad. Emerg. Med..

[10] Vargo J.J., Zuccaro G., Dumot J.A., Conwell D.L., Morrow J.B., Shay S.S. (2002). Automated graphic assessment of respiratory activity is superior to pulse oximetry and visual assessment for the detection of early respiratory depression during therapeutic upper endoscopy. Gastrointest. Endosc..

[11] Waugh J.B., Epps C.A., Khodneva Y.A. (2011). Capnography enhances surveillance of respiratory events during procedural sedation: A meta-analysis. J. Clin. Anesth..

[12] Long B., Koyfman A., Vivirito M.A. (2017). Capnography in the Emergency Department: A Review of Uses, Waveforms, and Limitations. J. Emerg. Med..

[13] Selby S.T., Abramo T., Hobart-Porter N. (2018). An Update on End-Tidal CO2 Monitoring. Pediatr. Emerg. Care.

[14] Langhan M.L., Chen L. (2008). Current utilization of continuous end-tidal carbon dioxide monitoring in pediatric emergency departments. Pediatr. Emerg. Care.

[15] Langhan M.L., Shabanova V., Li F.Y., Bernstein S.L., Shapiro E.D. (2015). A randomized controlled trial of capnography during sedation in a pediatric emergency setting. Am. J. Emerg. Med..

[16] Aslan N., Yildizdas D., Horoz O.O., Arslan D., Coban Y., Sertdemir Y. (2020). Effects of Sedation and/or Sedation/Analgesic Drugs Administered during Central Venous Catheterization on the Level of End-tidal Carbon Dioxide Measured by Nasal Cannula in Our PICU. Indian J. Crit. Care Med..

[17] Yldzdaş D., Yapcoǧlu H., Ylmaz H.L. (2004). The value of capnography during sedation or sedation/analgesia in pediatric minor procedures. Pediatr. Emerg. Care.

[18] Cacho G., Pérez-Calle J.L., Barbado A., Lledó J.L., Ojea R., Fernández-Rodríguez C.M. (2010). Capnography is superior to pulse oximetry for the detection of respiratory depression during colonoscopy. Rev. Española Enfermedades Dig..

[19] Conway A., Douglas C., Sutherland J. (2015). Capnography monitoring during procedural sedation and analgesia: A systematic review protocol. Syst. Rev..

[20] Damam S., Meshram R.J., Taksande A., Lohiya S., Khurana A., Patel A., Khandelwal R., Nath R., Javvaji C.K., Kakkat S. (2024). Navigating Pediatric Capnography: A Comprehensive Review of Scope and Limitations. Cureus J. Med. Sci..

[21] Anderson J.L., Junkins E., Pribble C., Guenther E. (2007). Capnography and depth of sedation during propofol sedation in children. Ann. Emerg. Med..

[22] Deitch K., Miner J., Chudnofsky C.R., Dominici P., Latta D. (2010). Does end tidal CO2 monitoring during emergency department procedural sedation and analgesia with propofol decrease the incidence of hypoxic events? A randomized, controlled trial. Ann. Emerg. Med..

[23] Cambra Lasaosa F.J., Pons Ódena M. (2003). Pulsioximetría y capnografía. An. Pediatría.

[24] Sullivan K.J., Kissoon N., Goodwin S.R. (2005). End-tidal carbon dioxide monitoring in pediatric emergencies. Pediatr. Emerg. Care.

[25] Krauss B., Hess D.R. (2007). Capnography for procedural sedation and analgesia in the emergency department. Ann. Emerg. Med..

[26] Sahyoun C., Cantais A., Gervaix A., Bressan S., Löllgen R., Krauss B., Pediatric Emergency Medicine Comfort and Analgesia Research in Europe (PemCARE) group of the Research in European Pediatric Emergency Medicine. (2021). Pediatric procedural sedation and analgesia in the emergency department: Surveying the current European practice. Eur. J. Pediatr..

[27] Advanced Life Support Group, Advanced Life Support Group (2016). Introduction. Advanced Paediatric Life Support: A Practical Approach to Emergencies.

[28] Lee J.H., Ko H., Park J.B., Ji S.H., Kim J.T. (2025). Oxygen supplementation in pediatric sedation-prospective, multicenter, randomized controlled trial. Anesthesiology.

[29] Saunders R., Struys M.M.R.F., Pollock R.F., Mestek M., Lightdale J.R. (2017). Patient safety during procedural sedation using capnography monitoring: A systematic review and meta-analysis. BMJ Open..

[30] Nitzan M., Romem A., Koppel R. (2014). Pulse oximetry: Fundamentals and technology update. Med. Devices (Auckl. NZ).

[31] Bass J.L., Corwin M., Gozal D., Moore C., Nishida H., Parker S., Schonwald A., Wilker R.E., Stehle S., Kinane T.B. (2004). The effect of chronic or intermittent hypoxia on cognition in childhood: A review of the evidence. Pediatrics.

[32] Tolnai J., Rárosi F., Tóth I., Babik B., Novák Z., Peták F., Fodor G.H. (2024). Relationships between capnogram parameters by mainstream and sidestream techniques at different breathing frequencies. Sci. Rep..

